# Chemoprophylaxis with sporozoite immunization in *P*. *knowlesi* rhesus monkeys confers protection and elicits sporozoite-specific memory T cells in the liver

**DOI:** 10.1371/journal.pone.0171826

**Published:** 2017-02-09

**Authors:** Sathit Pichyangkul, Michele D. Spring, Kosol Yongvanitchit, Utaiwan Kum-Arb, Amporn Limsalakpetch, Rawiwan Im-Erbsin, Ratawan Ubalee, Pattaraporn Vanachayangkul, Edmond J. Remarque, Evelina Angov, Philip L. Smith, David L. Saunders

**Affiliations:** 1 Armed Forces Research Institute of Medical Sciences (AFRIMS), Bangkok, Thailand; 2 Biomedical Primate Research Centre, Rijswijk, The Netherlands; 3 Walter Reed Army Institute of Research, Silver Spring, Maryland, United States of America; Agency for Science, Technology and Research - Singapore Immunology Network, SINGAPORE

## Abstract

Whole malaria sporozoite vaccine regimens are promising new strategies, and some candidates have demonstrated high rates of durable clinical protection associated with memory T cell responses. Little is known about the anatomical distribution of memory T cells following whole sporozoite vaccines, and immunization of nonhuman primates can be used as a relevant model for humans. We conducted a chemoprophylaxis with sporozoite (CPS) immunization in *P*. *knowlesi* rhesus monkeys and challenged via mosquito bites. Half of CPS immunized animals developed complete protection, with a marked delay in parasitemia demonstrated in the other half. Antibody responses to whole sporozoites, CSP, and AMA1, but not CelTOS were detected. Peripheral blood T cell responses to whole sporozoites, but not CSP and AMA1 peptides were observed. Unlike peripheral blood, there was a high frequency of sporozoite-specific memory T cells observed in the liver and bone marrow. Interestingly, sporozoite-specific CD4^+^ and CD8^+^ memory T cells in the liver highly expressed chemokine receptors CCR5 and CXCR6, both of which are known for liver sinusoid homing. The majority of liver sporozoite-specific memory T cells expressed CD69, a phenotypic marker of tissue-resident memory (T_RM_) cells, which are well positioned to rapidly control liver-stage infection. Vaccine strategies that aim to elicit large number of liver T_RM_ cells may efficiently increase the efficacy and durability of response against pre-erythrocytic parasites.

## Introduction

After thirty years of vaccine research, the world’s first vaccine against malaria, known as RTS,S (brand name Mosquirix™ by GlaxoSmithKline), has recently been given a positive review by regulators with the European Medicines Agency (EMA) for use in young children aged 6 weeks to 17 months outside the European Union. Containing the C-terminus and repeat regions of the *Plasmodium falciparum* circumsporozoite protein (CSP) fused to the hepatitis B surface antigen, this vaccine could provide a significant contribution to reducing the burden of malaria on African children, despite not reaching the 75% efficacy target set by WHO’s Malaria Vaccine Technology Roadmap.

RTS,S vaccine elicits an antibody response against the repeat regions of CSP as well as CD4^+^, but not CD8^+^ T cell responses. Detailed analysis from phase 3 trials shows that anti-CSP antibody response does have some correlation with protection [1]. Decline of antibody levels was rapid over the first 6 months; this may explain why the vaccine elicits short-term protection and suggests that the protection could depend primarily on circulating antibodies. Cellular T cell responses to eliminate the liver phase are likely required for long-term, sterile protection. Efforts are ongoing to improve the magnitude, durability and also breadth of protective immune responses for the 2^nd^ generation malaria vaccines and include techniques such as using different dose regimen/schedules, alternative vaccine platforms and combination of RTS,S vaccine with other vaccine antigens of pre-erythrocytic, blood, and sexual stages.

Whole *P*. *falciparum* sporozoite vaccines including CPS and radiation-attenuated sporozoite (RAS) vaccines, consistently provide better protection and durability in controlled human malaria infection (CHMI) than RTS,S vaccine [2, 3]. Data generated from whole sporozoite vaccines in a murine model indicate that protection against pre-erythrocytic parasites requires both antibody and T cell responses, especially from liver CD8^+^ T cells that produce IFN-γ or directly kill infected liver cells by cell-cell contact [4–7]. The role of local tissue immunity has received more attention lately primarily due to the discovery of a new subset of memory T cells termed tissue-resident memory (T_RM_) cells. These long-lived and non-recirculating T_RM_ cells permanently reside in non-lymphoid tissues including skin, brain, vagina, and lung and provide rapid, effective and long-term local protection against reinfection relative to circulating counterpart memory T cells [8–12]. This novel memory T cell subset expresses CD103 (αEβ7 integrin) and CD69 (C-type lectin), both of which are involved in cell adhesion and tissue retention [13]. These T_RM_ cells express greater T cell receptor (TCR) affinity and secret IFN-γ faster than do circulating memory T cells [14, 15]. While long-term local immune protection by T_RM_ cells has been consistently documented in murine models of virus and bacterial infections including vaccinia virus, lymphocytic choriomeningitis virus, herpes simplex virus, influenza and tuberculosis [8–12], the role of T_RM_ cells against malaria pre-erythrocytic parasites has remained less defined.

Most analysis on T cell responses elicited by whole sporozoite vaccines has been generated from mouse models which do not necessarily replicate human responses. In addition, studies in humans have primarily focused on T cells isolated from peripheral blood which may not reflect activity in the liver where protective immune responses occur since ethical and practical limitations preclude T cell collection from the liver. An alternative model would be to use non-human primates (NHPs) to study local tissue immune responses. Rhesus monkeys are phylogenetically close to humans and similarities of T cell responses in both species have been reported [16, 17]. Intravenous immunization with irradiated *P*. *falciparum* sporozoites (PfSPZ) was used to model liver stage cellular responses in rhesus macaques, however T_RM_ cells in the rhesus liver have not yet been characterized [18, 19].

CPS immunization was first explored in a rodent model and showed complete protection against subsequent sporozoite challenge [20, 21]. A similar level of protection was later confirmed in humans, and the protection persisted for over 2 years [3, 22]. The advantage of using CPS immunization may be that full development of liver-stage parasites enables presentation of an array of liver parasite antigens to the host immune system, resulting in a broader immunity. This hypothesis is supported by a recent study which shows that protective immune responses generated from immunization with late-liver-stage arresting genetically attenuated sporozoites were greater than those generated from immunization with early-liver-stage arresting genetically attenuated sporozoites [23].

*P*. *knowlesi* is now recognized as the fifth human parasite [24] and human cases have been reported across Southeast Asia [25]. We have an established P. *knowlesi* rhesus monkey model for immunization and mosquito bite challenge at AFRIMS, and we conducted a pilot study evaluating if CPS immunization could afford sterile protection, and if so, to evaluate sporozoite-specific memory T cells in different tissue compartments. In this paper, we show that a CPS *P*. *knowlesi* vaccine induced protection in rhesus monkeys and report on the anatomical distribution and the homing phenotype of sporozoite-specific memory T cells.

## Materials and methods

### Ethics statement

Animals were housed and cared in a facility accredited by the Association for Assessment and Accreditation of Laboratory Animal Care, International (AAALAC), incompliance with the Animal Welfare Act and the eight edition, Guide for the Care and Use of Laboratory Animals (National Research Council, 2011) and in accordance with all applicable USDA, Office of Laboratory Animal Welfare and Department of Defense guidelines. All animal procedures were approved by The United States Army Medical Directorate-Armed Forces Research Medical Sciences (USAMD-AFRIMS) Institutional Animal Care and Use Committee (IACUC). Animals were housed individually in standard squeeze-type stainless steel cages with a minimum floor space of 6.4 square feet with two pieces of toys such as steel mirror, gnawing sticks, piece of wood branch, etc. During the experiment, the animals were retained in a social environment through visual and auditory and, where possible, tactile contact (in the form of double grid partitions) with other monkeys. The animal cages were cleaned daily and sanitized biweekly. Monkeys received complete the local commercial diet (The Perfect Companion.Co, Ltd, Thailand) supplemented with mixed fresh fruit or vegetables, treats, and vitamin C. Animals were fasted before procedures requiring anesthesia, but drinking water was offered ad libitum. All animal procedures such as blood collection and mosquito bite challenge were performed under anesthesia using ketamine hydrochloride (5–20 mg/kg, IM), and all efforts were made to minimize stress, improve housing conditions, and to provide enrichment opportunities. Animals were humanely euthanized at the study endpoint. The monkeys were sedated with ketamine hydrochloride injection. Once sedated, injectable commercial euthanasia agent (fatal-plus) were utilized for euthanasia in accordance with the American Veterinary Medical Association (AVMA) Guidelines for the Euthanasia of Animals (2013 Edition).

### *P*. *knowlesi*-infected mosquito preparation

*Anopheles dirus* mosquitoes were maintained in the insectary of the Entomology Department, AFRIMS, Bangkok, Thailand. *P*. *knowlesi*-infected mosquitoes were prepared as previously described [26]. The gametocytes required 14–16 days to complete the sexual cycle in mosquitos. Oocyst infection was determined by midgut dissection 7 days post feed, and sporozoite infection was rated by salivary gland dissection 13–14 days post feed (ratings: 0 = 0 spz; 1 = 1–10 spz; 2 = 11–100 spz; 3 = 101–1,000 spz; 4 = >1,000 spz). Pools of mosquitoes that developed sporozoite ratings of 4 were employed for both the immunization and the challenge experiment. Most of infected mosquitoes prepared in our laboratory had >100,000 sporozoites/mosquito.

### Animals, CPS immunization and challenge

**Fig 1:**
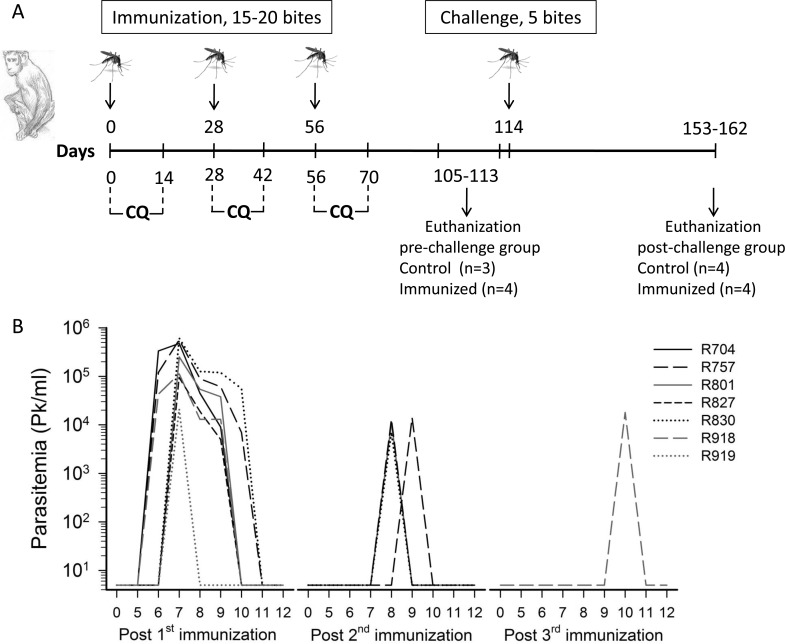
Experimental design and *P*. *knowlesi* parasitemia during CPS immunization. (A) Fifteen healthy naive rhesus macaques were administered 3 sequences of bites from 15–20 *P*. *knowlesi*-infected *Anopheles dirus* mosquitoes (immunized group; n = 8) or uninfected mosquitoes (control group; n = 7) on days 0, 28 and 56. Each immunization was followed by 14 days of daily oral chloroquine (CQ) treatment at a dose of 10 mg/kg/day in order to kill blood parasites at trophozoite stage. Seven animals (4 immunized /3 controls) were sacrificed between days 105–113 for evaluation of pre-challenge immune responses, while eight animals (4 immunized /4 controls) were sacrificed between days 153–162 (days 39–48 post challenge). (B) Transient blood-stage *P*. *knowlesi* parasitemia in seven of eight animals in the experimental group during CPS immunization.

Schedule of immunization and challenge was shown in Fig 1A. Fifteen healthy naïve rhesus monkeys (age 5–13 years old) were randomly divided into 2 groups in this pilot study. Animals in an immunized group (n = 8) received three immunizations on the middle of abdomen at days 0, 28 and 56. Each immunization consisted of 15–20 bites by *P*. *knowlesi*-infected mosquitoes under chloroquine (CQ) treatment. Drug treatment started on the same day as sporozoite inoculation with oral administration of CQ (at a dose of 10 mg base/kg once daily) for 14 consecutive days in order to kill blood parasites at trophozoite stage. Animals in a control group (n = 7) received 15–20 bites by non-infected mosquitoes under the same drug treatment regimen.

Starting from the 4^th^ day of each CQ treatment, blood smears were daily examined for parasites until day 12 by thick and thin blood smear microscopy. After the last dose of CQ treatment at day 70, blood samples were collected daily until day 89 for measurement of CQ levels. After 3 immunizations were completed, 4 animals in each group were challenged via 5 bites of infected-mosquitoes at day 114 (58 days after the last vaccine dose). Starting four days after challenge, daily blood collections were taken for assessment of blood stage parasite infection. When the parasitemia level reached 0.2–2%, the monkeys were immediately treated with artesunate (IV, 8 mg/kg), followed by seven consecutive days of quinine dihydrochloride (IM, 20 mg/kg), twice daily.

### Tissue collection and cell separation

To evaluate memory T cell response, 4 of immunized animals and 3 control animals were euthanized between days 105–113 (“pre-challenge”) and then the rest of animals in each group were euthanized between days 153–162 (39–48 days post-challenge) (Fig 1A). Peripheral blood was collected at baseline, 14 days after each vaccination and before euthanasia, and then processed for PBMC isolation by centrifugation using Histopaque-1077 (Sigma-Aldrich, St. Louis, MO). The liver, spleen, bone marrow, axillary lymph nodes, inguinal lymph nodes and mesenteric lymph nodes were collected immediately after euthanasia. The spleen, bone marrow and lymph nodes were homogenized between the frosted ends of two slides whereas the liver tissues were cut into small pieces and homogenized in the presence of 1 μg/ml of collagenase type 1 (Life Technologies, Grand Island, NY) using automated tissue dissociation (MiltenyiBiotec, Auburn, CA) and further incubated at 37 C for another 1 h. Digested liver cells and cells derived from the spleen, bone marrow and lymph nodes were then passed through 70 μm cell strainers (BD Falcon, Durham NC). Then, single cell suspensions were centrifuged using Histopaque-1077 to obtain mononuclear immune cells. Contaminated red blood cells were removed by treating with ammonium chloride-based lysing solution (BD Biosciences, San Jose, CA). PBMCs and mononuclear immune cells derived from different tissues were frozen in liquid nitrogen until use.

### Antibody response

Antibody response against whole sporozoites was assessed by immunofluorescence assay (IFA). Separate multiwell slides were coated with *P*. *knowlesi* sporozoites (10,000 sporozoites/well), air dried, and fixed with acetone. Slides were blocked with 1% bovine serum albumin (BSA) in PBS (PBS-BSA) for 30 min at room temperature. Serum, diluted in PBS-BSA, was added to the wells, and the slides were incubated in a humidified chamber for 1 h at room temperature. The slides were washed with PBS, and fluorescein isothiocyanate-labeled goat anti-monkey IgG antibody (Sigma-Aldrich) was added for 30 min at room temperature. Slides were washed, mounted in Fluoromount, and viewed with an Olympus microscope. The IFA titer was defined as the last dilution at which fluorescence intensity was higher than that of baseline serum.

Titers against CSP, cell traversal protein for ookinetes and sporozoites (CelTOS) and apical membrane antigen 1 (AMA1) antigens were measured by enzyme-linked immunosorbent assay (ELISA). Briefly, 96-well ELISA plates (Dynex, Chantilly, VA) were coated with recombinant *P*. *knowlesi* CSP, CelTOS and AMA1 (domain I-II proteins) (0.5 μg/well) at 4 C overnight, then washed with PBS containing 0.1% Tween 20 (Sigma-Aldrich). After blocking with 1% BSA in PBS with 0.1% Tween 20, serum samples in serial 2 fold dilutions were added into coated, blocked plates and incubated for 2 h. Antibodies specific to each antigen were detected by anti-monkeys IgG (Sigma-Aldrich) conjugated with peroxidase and developed with substrate using equal parts of solution A (2,2’-azino-di-(3-ethylbenzthiazoline-6-sulfonate)) and B (H_2_O_2_) (KPL). The reaction was stopped with 5% sodium dodecyl sulfate stop solution and the plates were read at 405 nm on a SPECTRAmax plate reader (Molecular Devices Inc.). The antibody titer was defined as that serum dilution giving an optical density of 1.0 in our assay.

### Intracellular cytokine staining

T cell responses were assessed by intracellular cytokine staining (ICS). Cryopreserved PBMCs and mononuclear immune cells (1x10^6^ cells in 200 ul of complete medium) from different tissue compartments were stimulated for 16 h with cryopreserved 100,000 *P*. *knowlesi* sporozoites (low mitogenic activity), CSP peptides (88 15–mer peptide overlapping by 11 amino acids), or AMA1 peptides (138 15–mer peptide overlapping by 11 amino acids) at a final concentration of each peptide of 1 ug/ml. All stimulated cell cultures contained 1 μg/ml of anti-CD28 (clone L293, BD Biosciences) and 1 μg/ml of anti-CD49 (clone L25, BD Biosciences). Three samples per tissue/blood sample were used: 1) mononuclear immune cells cultured with medium served as an unstimulated control (background); 2) mononuclear immune cells stimulated with superantigen staphylococcal enterotoxin B (SEB) (4 μg/ml) served as a positive control; and 3) mononuclear immune cells stimulated with 100,000 *P*. *knowlesi* sporozoites served as an experimental sample.

For the last 6 h of sporozoite stimulation and the last 14 h of peptide stimulation, Golgiplug was added to inhibit cytokine secretion. Then cells were washed and stained with a panel of antibodies specific for surface markers, including anti-CD3 (clone SP34, BD Biosciences), anti-CD4 (clone L200, BD Biosciences), anti-CD8 (clone SK1, BD Biosciences), anti-CD16 (clone 3G8, BD Bioscience), anti-TCR γδ (clone B1, BioLegend, San Diego CA), anti-CD45RA (clone DX2, BioLegend), anti-CCR7 (clone 150503, R&D System), anti-CD103 (clone B-Ly7, eBioscience, San Diego CA), anti-CXCR3 (clone 1C6/CXCR3, BioLegend), anti-CCR5 (clone 3A9, BioLegend) and CXCR6 (clone 56811, R&D). The stained cells were fixed/permeabilized and intracellular cytokine was stained with monoclonal antibodies against IFN-γ (clone B 27, BioLegend). Finally, stained cells were analyzed by 6-color flow cytometry (BDFACSCanto, BD Biosciences). T cell populations were analyzed by excluding CD16^+^ NK and γδ T cells. The values of unstimulated controls (background) were deducted from sporozoite-specific T cell responses before being reported.

To evaluate the role of CD69 positive memory T cells in cytokine responses, mononuclear immune cells from the liver, spleen and bone marrow were stained with anti-CD69 (clone FN50, BD Biosciences) and CD69 positive cells were depleted by fluorescence-activated cell sorter (FACSAria III, BD Biosciences). CD69 depleted mononuclear immune cells were stimulated with sporozoites and assessed for IFN-γ production compared with the non-depleted population.

### Statistical analysis

The data were analyzed using SPSS 12.0 for Windows (SPSS Inc., Chicago, IL). Antibody titers were log transformed before testing of differences. Differences in antibody and T cell responses were analyzed by using the Friedman test and the Wilcoxon Signed Rank test (for non-normally distributed data), with the Bonferroni correction for multiple comparisons. P values of <0.05 were considered statistically significant.

## Results

### CPS immunization in *P*. *knowlesi* rhesus monkey model

Eight healthy naïve animals received three CPS immunizations at days 0, 28 and 56 with *P*. *knowlesi* sporozoites (15–20 bites of infected mosquitoes) and seven healthy naïve control animals received 15–20 bites of non-infected mosquitoes under daily oral CQ chemoprophylaxis for 14 days (Fig 1A). No blood parasitemia was detected microscopically in control animals after each of the three immunizations; however, a transient blood parasitemia was detected in all but one immunized animals during days 6 through10 following the first immunization (Fig 1B). The number of blood-stage parasites (range 7,000–615,000 parasites/ml) after the first immunization was higher than those after the second and third immunizations (range 7,000–14,000 parasite/ml). Three immunized animals (R704, R827 and R919) developed transient blood parasitemia after the first immunization only, another three (R757, R830 and R801) were parasitemic after the first and second, one (R918) developed a parasitemia after all three immunizations and one animal (914) remained aparasitemic throughout (Fig 1B).

### CPS immunization elicits protection from *P*. *knowlesi* sporozoite bite challenge

**Fig 2:**
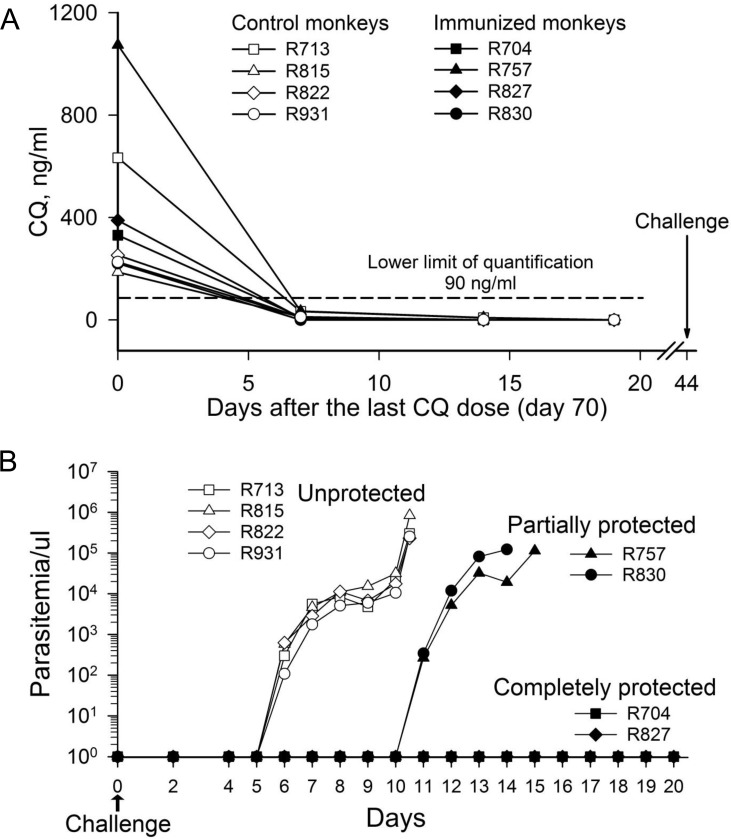
Residual CQ levels and malaria patency post-challenge. (A) Plasma CQ levels dropped below the lower limit of quantification by day 7 following administration in animals in the immunized group (n = 4) and control group (n = 4). (B) Blood-stage *P*. *knowlesi* parasitemia measured by light microscopy following challenge from the bites of 5 infected mosquitoes at day 114. Animals who developed malaria patency were treated with artesunate and quinine dihydrochloride once parasitemia reached 0.2–2%.

Since the residual levels of CQ could interfere with sporozoite challenge outcomes, we measured the drug plasma levels of 8 animals that went on to challenge. As depicted in Fig 2A, the CQ plasma levels reached baseline (<90 ng/ml of CQ) within seven days of the last immunization session. To assess if CPS immunization induces protective effcacy, four CPS immunized animals and four control animals then underwent sporozoite challenge via 5 bites of *P*. *knowlesi*-infected mosquitoes 58 days after the last immunization (day 114) (Fig 1A). Protection was defined as negative blood parasitemia microscopy until day 20 after sporozoite challenge. All four control animals developed patent parasitemia at day 6 after sporozoite challenge. Two of four animals that received CPS immunization were completely protected; two had a marked delay in patency, developing blood parasitemia at day 11, five days later compared to control animals (Fig 2B). The CPS immunized monkeys that developed transient blood-stage parasitemia only after the first immunization (R704 and R827) demonstrated complete protection whereas those that developed transient blood-stage parasitemia after the first and second immunization (R757 and R830) demonstrated partial protection.

### CPS immunization induces pre-erythrocytic immunity against *P*. *knowlesi*

**Fig 3:**
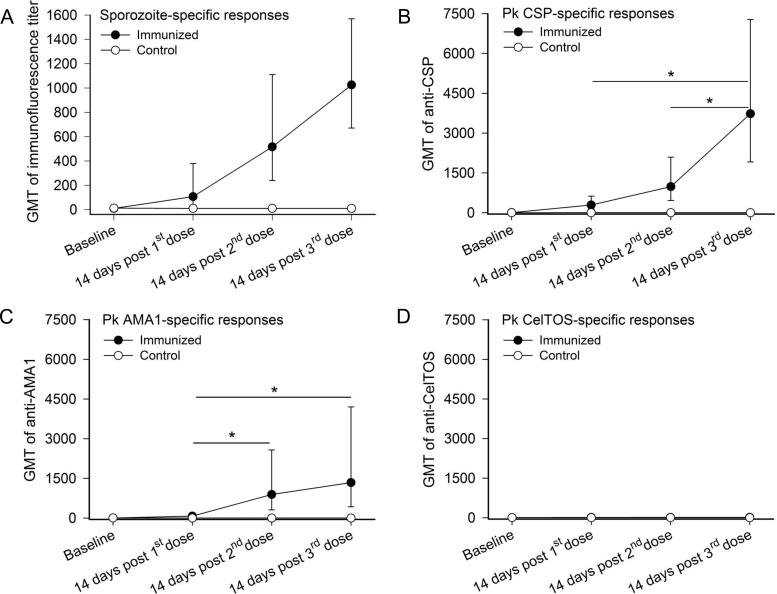
Kinetics of serum antibody responses. Measurement of antibody responses was conducted from serum of control animals (n = 7) and immunized animals (n = 8). Serum antibody responses to (A) *P*. *knowlesi* sporozoites measured by IFA, (B) CSP measured by ELISA, (C) AMA1 measured by ELISA and (D) CelTOS measured by ELISA. Each data point represents the GMT± SE (*p < 0.05, Bonferroni correction).

All CPS immunized animals developed antibody responses after immunization. At day 14 after the first immunization, geometric mean titer (GMT) of antibodies to whole sporozoites measured by IFA were 107 (95% CI = 30 to 380), and these continued to increase, peaking at 14 days after the last immunization with GMT IFA titers of 1,026 (95% CI = 670 to1,569) (Fig 3A). Serum antibody responses to *P*. *knowlesi* pre-erythrocytic antigens including CSP, AMA1 and CelTOS were also measured by ELISA. After immunization, animals developed antibody titers against CSP (peak GMT = 3,733; 95% CI = 1,914 to 7,281) and AMA1 (peak GMT = 1,343; 95% CI = 429 to 4,203) (Fig 3B and 3C). Immunized animals did not develop serum antibody response specific to CelTOS antigen (Fig 3D). No antibody responses to these antigens were detected in control animals.

**Fig 4:**
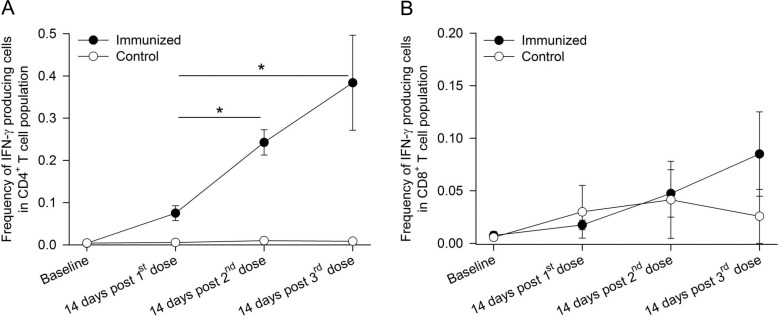
Kinetics of peripheral blood T cell responses. Peripheral blood T cell cytokine responses by ICS assay for (A) CD4^+^ and (B) CD8^+^ T cells against *P*. *knowlesi* sporozoites were measured in control animals (n = 7) and immunized animals (n = 8). Data shown are means ± SE of IFN-γ producing cells in the CD4^+^ or CD8^+^ T cell population (*p < 0.05, Bonferroni correction).

We next evaluated the kinetics of peripheral blood CD4^+^ and CD8^+^ T cell responses by measuring the frequency of IFN-γ producing cells by intracellular cytokine staining (ICS). As shown in Fig 4A, whole sporozoite-specific IFN-γ production in peripheral blood CD4^+^ T cells was detected after the first immunization and continued to increase after the second and third immunization (peak mean frequency of cytokine-producing CD4^+^ T cells ± SE = 0.4 ± 0.1%). Peak peripheral blood CD8^+^ T cell responses in the CPS immunized group were detected after the last immunization with the mean frequency of cytokine-producing CD8^+^ T cells ± SE = 0.09 ± 0.04% (Fig 4B). High background CD8^+^ T cell response was detected in control animals. Peak frequency of sporozoite-specific CD4^+^ T cells was about 4 fold higher than that of CD8^+^ T cells (p = 0.008, Wilcoxon Signed Ranks Test). After the third immunization (day 70), the frequencies of sporozoite-specific CD4^+^ and CD8^+^ T cells in completely protected animals (R704 and R827) were 2–15 fold higher than those in partially protected animals (R757 and R830), whereas no differences in antibody responses between the two groups were detected (data not shown).

Since CPS immunized animals developed antibody responses against CSP and AMA1, we next assessed T cell responses against *P*. *knowlesi* CSP and AMA1 peptides after the last immunization. PBMCs from 2 animals (14 days after last immunization) and spleen mononuclear immune cells from 4 animals (1 pre-challenge and 3 post challenge animals) that showed positive sporozoite-specific T cell responses were used to assess the reactivity against CSP and AMA1 peptides. Spleen cells showed much higher responses than PBMCs, allowing for clearer differentiation between positive and negative antigen reactivity. Results in S1 Fig demonstrate negligible CD4^+^ and CD8^+^ T cell responses specific to CSP and AMA1.

### Anatomical distribution of sporozoite-specific memory T cells, memory phenotype and homing receptor of T cells

**Fig 5:**
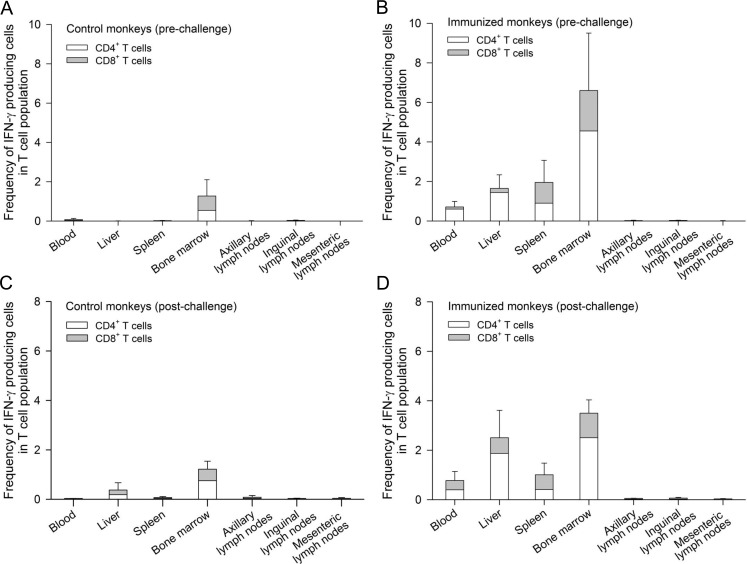
Anatomical distribution of sporozoite-specific memory CD4^+^ plus CD8^+^ T cells. The frequencies of *P*. *knowlesi* sporozoite-specific IFN-γ producing T cells isolated from tissue compartments from euthanized animals at pre-challenge in A) control animals (n = 3) and B) immunized animals (n = 4), and 39–46 days post challenge in a second cohort of C) control animals (n = 4) and D) immunized animals (n = 4). Data shown are means ± SE.

We then assessed the tissue distribution of sporozoite-specific memory T cell responses following CPS immunization. Immune cells were isolated from peripheral blood, liver, spleen, bone marrow, axillary, inguinal and mesenteric lymph nodes from two cohorts of animals (pre- and post–challenge). In pre-challenge animals (R801, R914, R918 and R919), high frequencies of sporozoite-specific memory T cells were observed in the bone marrow (total frequency of 6.6%; CD4^+^ T cells = 4.6%, CD8^+^ T cells = 2.0%), followed by spleen (total frequency of 1.9%; CD4^+^ T cells = 1.0%, CD8^+^ T cells = 0.9%), liver (total frequency of 1.60%; CD4^+^ T cells = 1.4%, CD8^+^ T cells = 0.2%) and peripheral blood (total frequency of 0.7%; CD4^+^ T cells = 0.6%, CD8^+^ T cells = 0.1%) (Fig 5A and 5B). There were negligible sporozoite-specific memory T cells detected in axillary, inguinal or mesenteric lymph nodes. Animal R919 had one episode of transient parasitemia with very strong sporozoite-specific T cell responses in all tissue compartments. Animal R918 had three, and R801 had two episodes of transient parasitemia and both animals had moderate T cell responses. Animal R914 had no transient pararasitemia during any of three CPS immunizations and had low T cell responses. One could speculate that the reason why animal R914 did not develop any transient parasitemia may be due to a very effective innate immunity via type I IFN response that was able to prevent liver-stage infection [27, 28].

In post-challenge animals (R704, R757, R827 and R830), while the frequencies of sporozoite-specific memory T cells in the peripheral blood, spleen and bone marrow either remained the same or declined (1–2 fold), there was an overall 0.6-fold increase in liver sporozoite-specific memory T cells compared to pre-challenge (Fig 5B and 5D).

Despite small numbers of animals in each group post-challenge, completely protected animals (R704 and R827) tend to have high frequencies of sporozoite-specific memory T cells in peripheral blood, liver, spleen and bone marrow compared to those partially protected (R757 and R830) and control animals (n = 4). Higher antibody levels against *P*. *knowlesi* sporozoites and CSP, but not AMA1 or CelTOS were also observed (S2 Fig).

**Fig 6:**
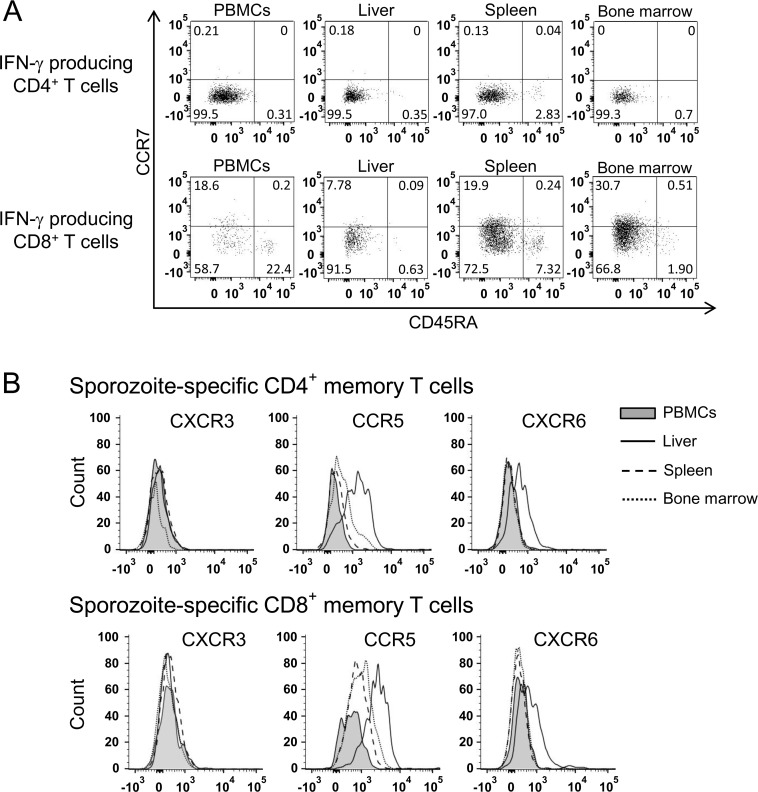
Memory phenotype and chemokine receptor expression of *P*. *knowlesi* sporozoite-specific T cells. Expression of central memory (CD45RA^-^ CCR7^+^), effector memory (CD45RA^-^ CCR7^-^) and terminal effector (CD45RA^+^ CCR7^-^) T cell markers on sporozoite-specific memory T cells isolated from different compartments (A). IFN-γ producing CD4^+^ and IFN-γ producing CD8^+^ T cells were gated and then analyzed for the expression of CCR7 and CD45RA. Data are representative of two animals (R704 and R827). CXCR3, CCR5 and CXCR6 expression by *P*. *knowlesi* sporozoite-specific memory T cells isolated from different compartments (B). IFN-γ producing CD4^+^ and IFN-γ producing CD8^+^ T cells were gated and then analyzed for the expression of CXCR3, CCR5 and CXCR6. Data are representative of three animals (R704, R827 and R919).

We assessed memory phenotype and chemokine homing receptors of T cells isolated from one pre-challenge animal (R919) and two post-challenge animals (R704 and R827) demonstrating high magnitude of sporozoite-specific T cell responses. The majority of sporozoite-specific memory CD4^+^ and CD8^+^ T cells in peripheral blood, liver, spleen and bone marrow of animals R704 and R827 had effector memory phenotype (T_EM_) as indicated by expression of CD45RA^-^ CCR7^-^ (Fig 6A and S3 Fig). Migration of memory T cells and homing to tissue is chemokine dependent. We next compared CXCR3, CCR5 and CXCR6 chemokine receptor expression on sporozoite-specific memory T cells in peripheral blood, liver, spleen and bone marrow of animals R704, R827 and R919. As depicted in Fig 6B and S3 Fig, increased expression of CCR5 and CXCR6 were consistently detected on both sporozoite-specific CD4^+^ and CD8^+^ memory T cells in the liver compared to those in other tissues. There was also a modest to moderate increase in CCR5 but not CXCR6 expression on sporozoite-specific memory CD4^+^ and CD8^+^ T cells in the spleen and bone marrow.

### Sporozoite-specific memory T cells expressed phenotypic markers of T_RM_

**Fig 7:**
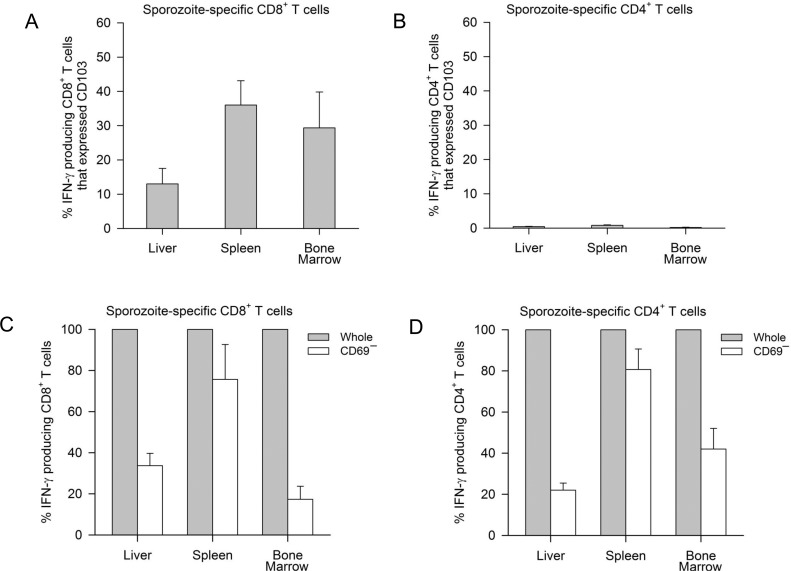
Phenotypic T_RM_ cell marker expression on *P*. *knowlesi* sporozoite-specific memory T cells. Percentage of *P*. *knowlesi* sporozoite-specific IFN-γ producing (A) CD8^+^ and (B) CD4^+^ T memory T cells that expressed CD103. IFN-γ response in *P*. *knowlesi* sporozoite-specific (C) CD8^+^ and (D) CD4^+^ memory T cells following depletion of CD69-positive cells. Mean ± SE of 3 animals (R704, R827 and R919) are shown.

Cell surface marker expression of CD103 and CD69 characteristic of T_RM_ cells was also assessed in 3 animals (R704, R827 and R919) that had high sporozoite-specific T cell responses. CD103 expression was higher in bone marrow (30%) and splenic (36%) sporozoite-specific memory CD8^+^ T cells compared to only 13% in the liver (Fig 7A), with negligible expression on CD4^+^ T cells (Fig 7B). Direct assessment of CD 69 expression is not possible, since *in vitro* stimulation with antigen leads to CD69 expression on all memory T cells [29]. We therefore conducted a CD69 cell depletion experiment to evaluate the proportion of antigen-specific memory T cells expressing CD69 in studied tissues. Flow cytometry analysis demonstrated IFN-γ producing CD8^+^ T cells were reduced to 34% in the liver, 17% in bone marrow and 76% in the spleen when CD69^+^ T cells were depleted (Fig 7C), implying that 66% of liver, 83% of bone marrow and only 24% of splenic sporozoite-specific memory CD8^+^ T cells expressed CD69. Similarly, a substantial proportion of sporozoite-specific memory CD4^+^ T cells expressed CD69 in the liver (78%) and bone marrow (58%), but only 19% in spleen (Fig 7D).

## Discussion

Evaluation of immune responses against malaria parasites in humans remains limited to sampling of peripheral blood, therefore animal models are often employed to provide more detailed and tissue-specific immunological studies to inform human malaria vaccine development. Non-human primate studies are limited by differences in the immunological response to primate-specific malaria infection and the lack of a robust challenge model for *P*. *falciparum* malaria. *P*. *knowlesi’*s ability to infect both humans and macaques with similar clinical responses transcends those limitations. Using a CPS immunization approach in rhesus monkeys, we demonstrated significant though incomplete protection against subsequent *P*. *knowlesi* challenge with sterile protection in two of four animals immunized. Further, we found that CPS immunization generated a higher liver tissue-tropic sporozoite-specific memory T cell response than that in peripheral blood, confirming prior observations in rhesus monkeys receiving immunization with PfSPZ [18]. Moreover, isolated sporozoite-specific memory T cells had T_EM_ phenotype and produced IFN-γ, known to inhibit the growth of liver-stage parasites [4, 5, 30, 31].

Higher frequencies of sporozoite-specific CD4^+^ T cell than CD8^+^ T cells were consistently observed in the livers and bone marrows. These findings were different from the two previous studies that showed higher frequencies of CD8^+^ T cell responses in the livers of immunized rhesus monkeys as compared to CD4^+^ T cell responses [18, 19]. The discrepancy between our findings and what were reported in those studies could be due to several factors; 1) Infecting malaria species. We used *P*. *knowlesi* which infects and causes malaria in rhesus monkeys, but it also can infect humans as well. The other two studies used *P*. *falciparum*, a human malaria parasite, in rhesus monkeys; 2) Experimental design. We immunized animals with live *P*. *knowlesi* sporozoite via 15–20 bites of infected mosquitoes under CQ treatment, while the other two studies immunized animals with irradiated *P*. *falciparum* sporozoites (1.35–2.7 x 10^5^ sporozoites) via intravenous administration and 3) Antigens used for recall assay. We used *P*. *knowlesi* sporozoites throughout the study for both CD4^+^ and CD8^+^ T cell assays, while one of the previous studies used *P*. *falciparum*-infected red blood cells as a recall antigen to obtain CD8^+^ T cell responses and irradiated sporozoites as a recall antigen to obtain CD4^+^ T cell responses [19].

Despite the important role of liver T cells in protective immunity against liver-stage parasites, there has been limited evidence to date regarding the homing phenotype of liver-derived sporozoite-specific memory T cells. Expression of chemokine receptors CXCR3, CCR5 and CXCR6 is known to regulate T cell migration to the liver [32]. In this study, we identified *P*. *knowlesi* sporozoite-specific liver memory T cells expressing chemokine receptors CCR5 and CXCR6 but not CXCR3. Specific ligands for CCR5 (CCL3 and CCL5) and CXCR6 (CXCL16) have been shown to be constitutively expressed within the liver sinusoids and to be involved in tissue homing [33, 34]. We also found a large proportion of liver sporozoite-specific memory CD4^+^ and CD8^+^ T cells had the T_RM_ phenotype based on CD69 expression, a tissue retention marker [13]. We were unable to localize CD69^+^ T cells anatomically within the liver due to technical staining limitations. Another T_RM_ cell marker; CD103 (αEβ7 integrin) was minimally expressed by liver sporozoite-specific memory CD8^+^ T cells. CD4^+^ T_RM_ cells poorly express CD103 [35, 36] and accordingly, we detected negligible sporozoite-specific memory CD4^+^ T cells (<5%) expressing CD103 in liver, bone marrow or spleen (Fig 7B). Over all, these findings support recent observations suggesting that CD69 and CXCR6 expression is unique for liver-resident memory CD8^+^ T cells and may be responsible for long-term local protective immunity [37–39].

We hypothesize that the observed liver sporozoite-specific CD69^+^ T_RM_ cells preferentially reside in the liver sinusoids rather than in the liver parenchyma based on the following: 1) prior murine studies combining intravascular and tetramer staining identified liver-derived lymphocytic chorimeningitis virus-specific CD69^+^ T_RM_ cells in the liver sinusoids rather than parenchyma, in contrast to lung and kidney where virus-specific CD69^+^ T_RM_ cells reside in tissue parenchyma [40, 41]; 2) systemic depletion of CD4^+^ and CD8^+^ T cells by intravenous infusion with mAb greatly decreases protection in CPS immunized mice upon sporozoite challenge [21], suggesting liver sinusoid-resident memory T cells which are directly exposed to and killed by circulating mAb rather than those in the liver parenchyma are critical for protection; and 3) human liver-resident CD69^+^ CCR5^+^ CXCR6^+^ NK cells preferentially reside within the liver sinusoids via the engagement of CCR5 and CXCR6 and their ligands expressed within the liver sinusoids [33, 42]. Our hypothesis was recently confirmed by an imaging study in mice showing the localization of CD8^+^ T_RM_ cells within the liver sinusoids after receiving RAS vaccine [43]. Furthermore, depletion of these liver sinusoid-resident CD8^+^ memory T cells led to the loss of protection.

The small diameter of the sinusoids (10 um) combined with low velocity of sinusoidal blood flow [44], may provide an excellent niche for close contact between sporozoite-specific CD69^+^ T_RM_ cells and sinusoid-resident antigen presenting cells including dendritic cells, Kupffer cells and endothelial cells during reinfection leading to a rapid and effective memory T cell response. Unlike other organs, which require immune cell infiltration to eliminate infected cells, every hepatocyte in the liver is in direct contact with sinusoidal endothelia [5] and therefore, sinusoid CD69^+^ T_RM_ cells could intravascularly patrol and directly probe sporozoite antigens expressed on infected hepatocytes through the approximately 150 nm fenestrations typical of these endothelial cells [45, 46]. Recent data suggest that hepatic effector CD8^+^ T cells arrest within liver sinusoids, recognize antigens and kill virus-infected hepatocytes by extending cytoplasmic protrusions through endothelial fenestrae [46]. Our study was conducted using a small sample size and more studies with larger sample sizes are required to investigate the correlation of protection with the frequency of T_RM_ cells in the liver. Given the importance of T_RM_ cells in inducing protection in murine models against virus, bacterial and liver-stage malaria infections [8–12, 43], a better understanding of function and longevity of liver sporozoite-specific CD69^+^ T_RM_ cells could better guide malaria vaccine development.

The malaria-specific memory T cell response in the bone marrow has been poorly investigated. This study demonstrates for the first time that CPS immunization elicits sporozoite-specific memory T cells in the bone marrow, with a large proportion expressing CD69. Bone marrow provides an optimal niche for memory T cell homing via IL-7expressing stroma cells [47, 48]. Bone marrow could also serve as the initial site for T cell priming via bone marrow resident antigen presenting cells [49, 50]. It remains unclear if bone marrow sporozoite-specific memory T cells are derived from memory T cells generated at the bone marrow, generated from other lymphoid tissues and then migrate to the bone marrow via blood circulation or from both. The role of sporozoite-specific bone marrow memory T cells in protection against pre-erythrocytic parasites merits further study. It should be noted that the majority of sporozoite-specific memory T cells in the spleen lacked T_RM_ cell marker CD69 confirming recent observations in mice receiving RAS vaccine [38]. This may suggest that the spleen could serve as a major reservoir for recirculating sporozoite-specific memory T cells.

Chakravarty *et al*. [51] recently described robust T cell responses in auricular lymph nodes draining the ear where *P*. *yoelii* sporozoites were injected. The absence of sporozoite-specific T cell responses in the axillary, inguinal and mesenteric lymph nodes in our study could be explained by the low densities of *P*. *knowlesi* sporozoites injected into the abdominal skin during immunization may have not migrated to the lymph nodes selected. Alternatively, the immune response in the lymph nodes may have occurred closer to the time of immunization and subsequently declined to undetectable levels at the time of animal sacrifice (>50 days after final immunization).

CPS immunized animals developed serum antibody response to whole sporozoites. The antibodies elicited recognized pre-erythrocytic antigens CSP and AMA1 but not CelTOS.

Lack of antibody response against CelTOS in our study was not due to a technical problem since serum samples from CelTOS-immunized monkeys from a previous trial, were always used as positive control. It is possible that the method of immunization (by mosquito bite) might not provide enough CelTOS antigen exposure to generate antibody responses. Unlike CSP, which localizes on the surface of the sporozoite, CelTOS localizes within sporozoite [52]. CelTOS-specific antibody and T cell responses were detected in humans immunized with intravenous RAS vaccine [52]. T cell response against CelTOS was not evaluated in this study so additional assays would be required to see if CPS immunization can generate CelTOS-specific T cell response. It is interesting to note that greater antibody responses to AMA1 were detected in partially protected compared to completely protected animals. The reason for this observation is likely due to more frequent exposure to transient blood-stage parasitemia during immunization and that this response was further boosted by later exposure to blood-stage parasites after challenge. Two partially protected animals had 2 episodes of transient parasitemias.

Malaria sporozoites also express a sugar molecule termed α-gal (Galα1-3Galb1-4GlcNAc-R) that is also expressed by gut bacteria. A recent study indicates that high levels of antibody, particularly IgM against α-gal, play a role in malaria protection in both mice and humans [53]. We detected pre-existing IgM and IgG antibodies against α-gal in all animals, and CPS immunization did not enhance the levels of anti- α-gal antibodies (IgM and IgG) (data not shown). We suspect that the pre-existing antibodies could mask the α-gal epitope, thereby preventing antigen recognition by B cells. In addition, α-gal is a carbohydrate moiety which does not induce strong immune responses. The detected levels of anti- α-gal antibodies in each animal also did not correlate with protection (data not shown).

We did not investigate the immune responses against *P*. *knowlesi* blood-stage parasites. Immunized animals were also exposed to transient blood-stage parasites during immunization process, therefore blood-stage immunity may as well contribute to the observed protection [54].

A limitation of the current model was that it failed to generate complete protection in all immunized animals. Of note, the two completely protected animals (R704 and R827) developed a transient blood-stage infection after the first immunization only and that these animals had high sporozoite-specific antibody and T cell responses. The two monkeys (R757 and R830) who experienced a delay in patency (partial protection) had measurable blood-stage parasitemia two times (after the first and second immunization) and also had low sporozoite-specific immune responses. Strong immune responses were also observed in animal R919 that was euthanized at pre-challenge and had only one episode of transient parasitemia. Given the small number of animals, it’s not possible to draw a conclusion that transient parasitemia only after the first vaccination could be used as a marker to predict protection.

Given that the numbers of infected- mosquito bites used for immunization were carefully controlled, it is unclear whether the observed response was a sporozoite dose-dependent phenomenon, or whether immunogenetic differences between animals played a role. Further sporozoite dose titration within the model, as well as immunogenomic study of experimental animals would be important next steps to clarify these findings. If the latter were true, then it is possible that even a single CPS immunization in the first 2 animals might have prevented infection from later sporozoite challenge.

Liver-stage infection with *P*. *knowlesi* sporozoites under CPS immunization in non-human primates provides a novel opportunity to investigate memory T cell localization and compare tissue-specific responses. We demonstrate here that *P*. *knowlesi* CPS immunization is able to elicit protection from subsequent sporozoite challenge in a rhesus monkey model. Sporozoite-specific CD4^+^ and CD8^+^ memory T cells producing IFN-γ were generated with a large proportion residing in the liver and bone marrow. Sporozoite-specific memory T cells in the liver expressed CD69 and chemokine receptors CCR5 and CXCR6, suggesting effector T_RM_ cells resident in the liver sinusoids. Our findings from the *P*. *knowlesi* rhesus model are likely to be relevant to human malaria, and provide further insights into generating sporozoite-specific T cell responses in the liver where protection is needed.

## Supporting information

S1 Fig IFN-γ producing CD4^+^ and CD8^+^ T cells specific to whole *P*. *knowlesi* sporozoites, CSP or AMA1 peptides. PBMCs (n = 2) and spleen mononuclear immune cells (n = 4) were used to assess antigen reactivity. Data shown for PBMCs are means and for spleen cells are means ± SE.
(TIF)

S2 Fig Tissue-specific T cell and antibody responses post-challenge. (A) High frequencies of sporozoite-specific IFN-γ producing T cells were observed in protected animals compared to partially protected and control animals. (B) Antibody titers were higher in protected animals against *P*. *knowlesi* whole sporozoite and CSP antigen, but not AMA1 (higher in partially protected animals), or CelTOS (no response).
(TIF)

S3 Fig Memory phenotype and chemokine receptor expression of *P*. *knowlesi* sporozoite-specific T cells. (A) Memory phenotypes of sporozoite-specific IFN-γ producing CD4^+^ and CD8^+^ T cells in animals R704 and R827. (B) Expression of CXCR3, CCR5 and CXCR6 on sporozoite-specific IFN-γ producing CD4^+^ and CD8^+^ T cells in animals R704, R827 and R919. Data shown for T cell memory phenotypes are means and expression of chemokine receptors are means ± SE.
(TIF)
