# Supplementary figures and images for: Chemoprophylaxis with sporozoite immunization in P. knowlesi rhesus monkeys confers protection and elicits sporozoite-specific memory T cells in the liver
Source: PLoS One. 2017 Feb 9;12(2):e0171826. doi: 10.1371/journal.pone.0171826 (PMC5300246; doi:10.1371/journal.pone.0171826)

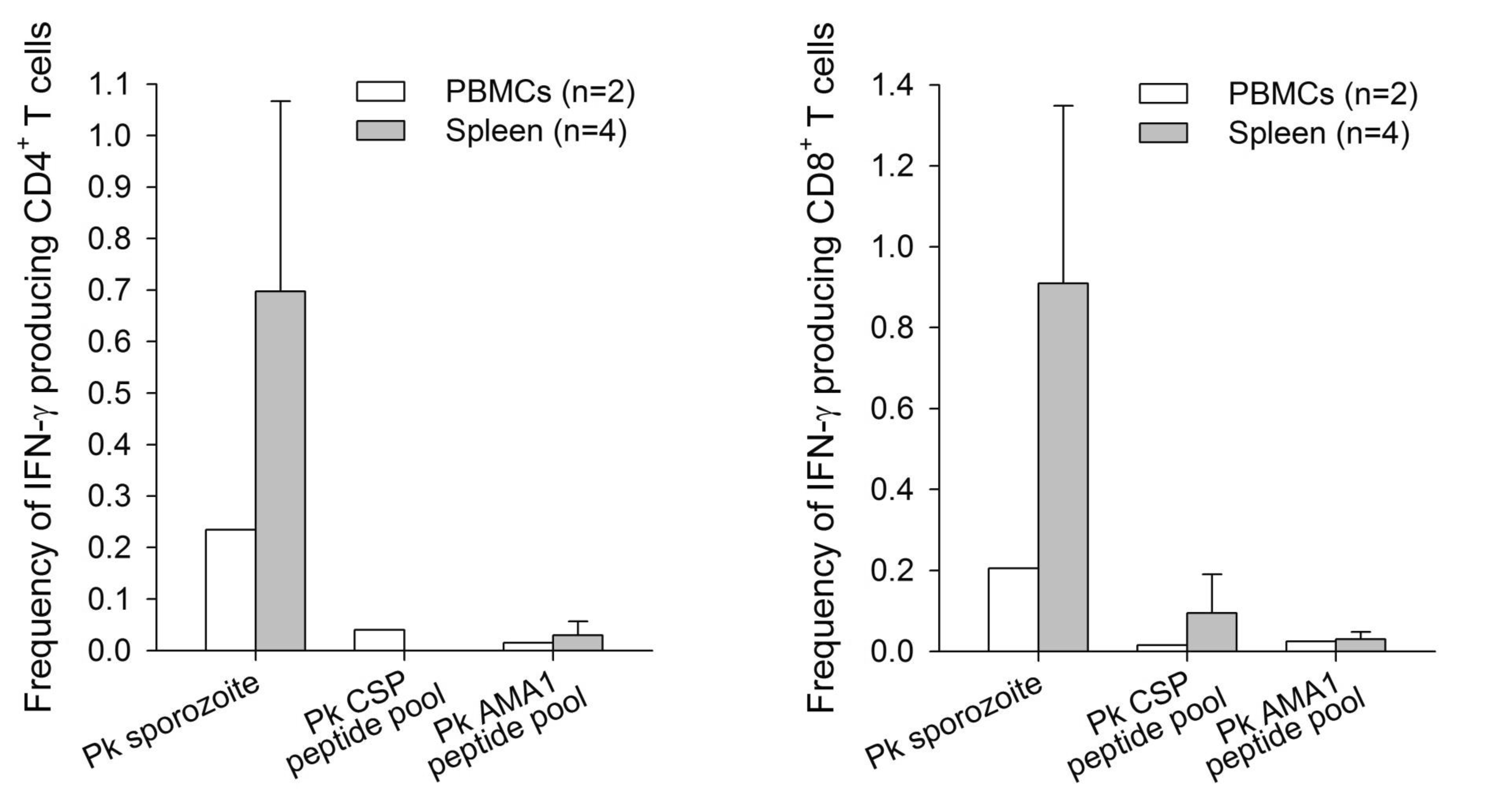

Supplement: S1 Fig — PBMCs (n = 2) and spleen mononuclear immune cells (n = 4) were used to assess antigen reactivity. Data shown for PBMCs are means and for spleen cells are means ± SE. (TIF) [file pone.0171826.s001.tif]

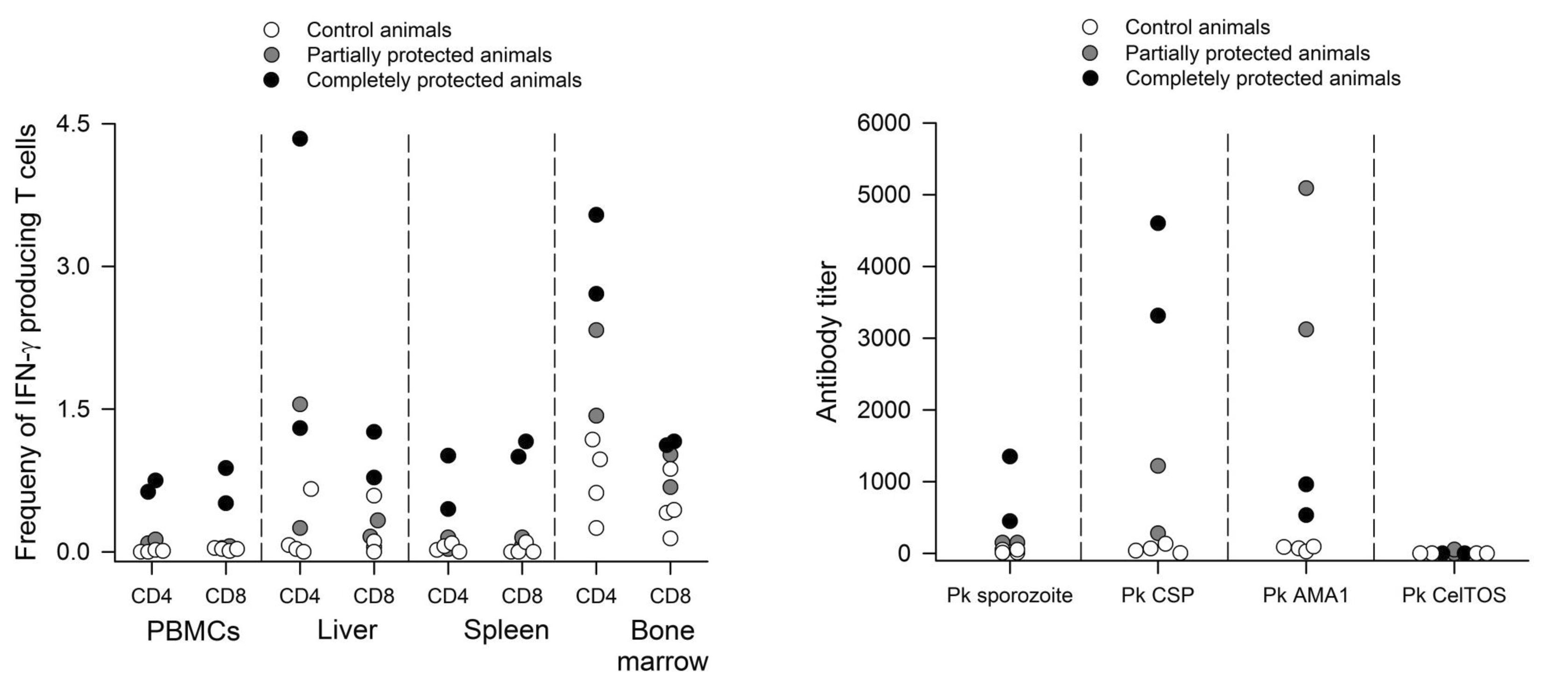

Supplement: S2 Fig — (A) High frequencies of sporozoite-specific IFN-γ producing T cells were observed in protected animals compared to partially protected and control animals. (B) Antibody titers were higher in protected animals against P. knowlesi whole sporozoite and CSP antigen, but not AMA1 (higher in partially protected animals), or CelTOS (no response). (TIF) [file pone.0171826.s002.tif]

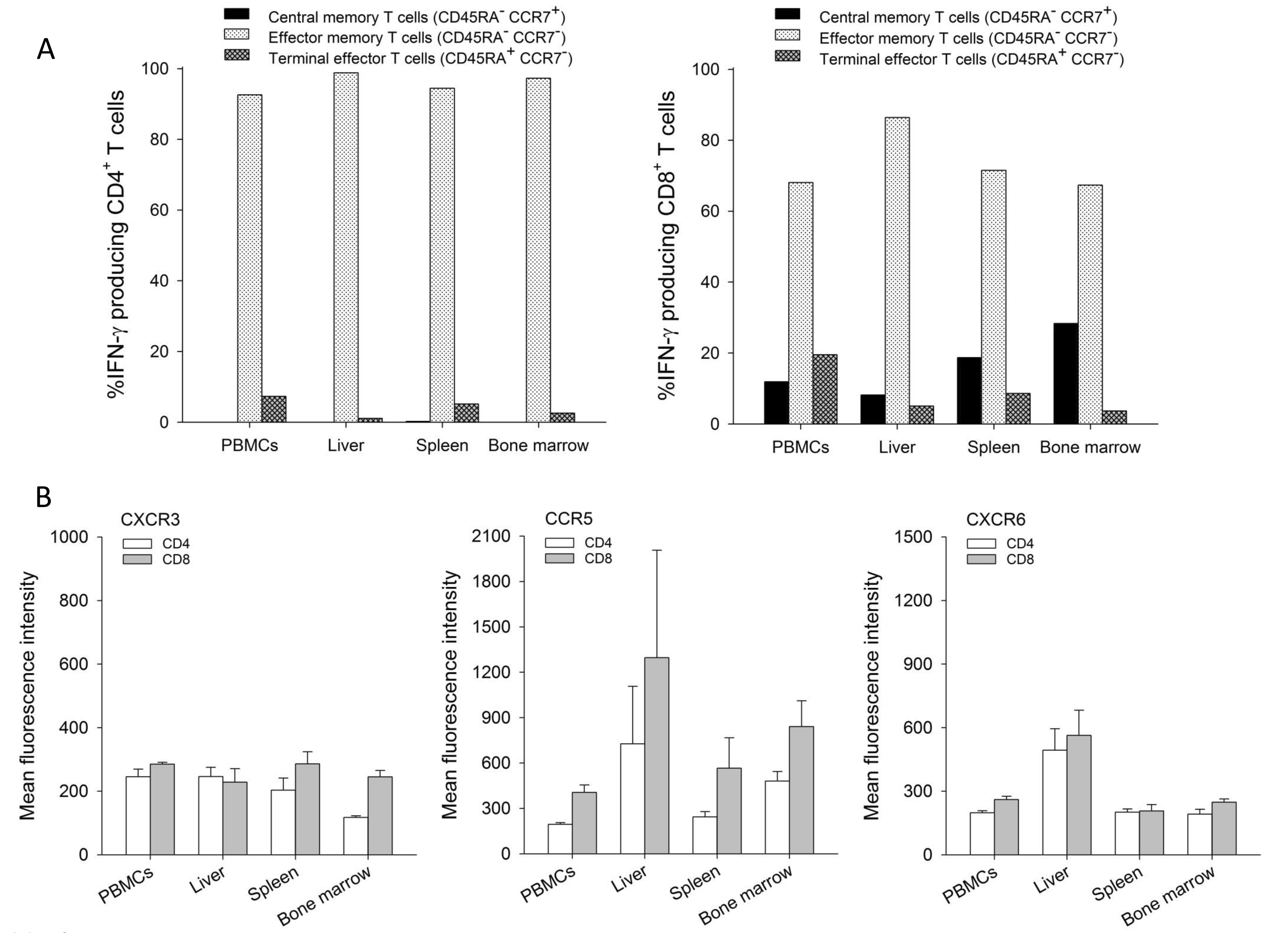

Supplement: S3 Fig — (A) Memory phenotypes of sporozoite-specific IFN-γ producing CD4+ and CD8+ T cells in animals R704 and R827. (B) Expression of CXCR3, CCR5 and CXCR6 on sporozoite-specific IFN-γ producing CD4+ and CD8+ T cells in animals R704, R827 and R919. Data shown for T cell memory phenotypes are means and expression of chemokine receptors are means ± SE. (TIF) [file pone.0171826.s003.tif]
